# The history of science in public: global audiences, local receptions*

**DOI:** 10.1590/S0104-59702026000100032

**Published:** 2026-08-14

**Authors:** James Poskett

**Affiliations:** i Associate professor, Department of History/University of Warwick. Warwick – United Kingdom. j.poskett@warwick.ac.uk

**Keywords:** Modern science, Horizons, Global history, History of science, Eurocentrism, Ciencia moderna, Horizontes, Historia global, Historia de la ciencia, Eurocentrismo

## Abstract

In responding to the historiographic review of *Horizons: a global history of science*, I address three core themes. First, the problem of “modern science.” Second, the nature of power in global history. Third, the importance of doing the history of science in public. I conclude by reflecting on the different cultures of public history in different parts of the world, as well as the challenge this poses.

## Resumen

En respuesta a la reseña historiográfica de Horizons: a global history of science, abordo tres temas principales. En primer lugar, el problema de la “ciencia moderna”. En segundo lugar, la naturaleza del poder en la historia global. En tercer lugar, la importancia de hacer historia de la ciencia en público. Concluyo con la reflexión de las diferentes culturas de la historia pública en distintas partes del mundo, así como sobre sus desafíos.

No author can control the reception of their work. Nor should they try to. Reception is one of the great pleasures – and, at times, frustrations – of writing and being read. That is multiplied many times over in the digital age, and again when your work appears in translation. It has been a real joy to see the diverse reception of *Horizons* over the past couple of years. I am especially grateful in this instance that the authors of the historiographic essay took the time to carefully read my book and provide a stimulating set of interventions. I hope to do equal justice in responding. Naturally, there are points of agreement and disagreement. But that’s all part of the fun of reception. To organise things, I have divided my response into three sections. First, the problem of “modern science.” Second, the politics of power and violence. And third, the history of science in public.

## The problem of “modern science”

What is *Horizons* actually a history of?^1^ This question gets to the heart of the more pointed critique of the reviewers. I found their discussion of the relationship between my narrative and Eurocentrism particularly engaging. By focusing on the problem of “modern science,” the reviewers highlight the lack of clarity surrounding the subject matter of the history of science. Much of this relates to how we write about knowledge systems beyond what the reviewers call “a traditional and universalizing Euro-North-American vision.” In more strident moments, the reviewers argue that this “narrative of knowledge production, which draws from so many regions, cultures, and contexts, assumes that they share the same vision of science.” And, perhaps most importantly, they argue that my history is really just the history of “the Euro-North-American validation of other knowledge systems.”

I find elements of agreement and disagreement here. In the first instance, I think it is important to recognise that *Horizons* is a global history of what I repeatedly refer to as “modern science.” I do not, however, take the term “modern science” as having much intrinsic content. It is not a natural kind. The historical category of “modern science” has nothing to do with truth, rationality, or a particular epistemic system. “Modern science” for me simply means the things people point to – for good reasons or otherwise – and call “science” today. Of course, as historians of science have argued for decades, what counts as “science” has changed and been contested in the past. But today, for reasons that *Horizons* itself makes clear, a relatively bounded set of knowledge systems is understood to constitute “science.” Writing the history of how we got to this position is not in my view teleological. Or at least, it is not teleological in an intellectually or politically suspect way. It is perfectly reasonable to write a history of how we got to where we are today, just as it is perfectly reasonable to write a history of roads not travelled.^2^ Historians of science – including myself – have been doing both for a long time. My first book was on a science that was ultimately rejected, for which the very term “pseudoscience” was coined (Poskett, 2019). *Horizons* is largely, but not exclusively, about sciences that were accepted and are now considered part of the core of “modern science.” That said, I also cover sciences such as astrology and eugenics precisely to demonstrate that the road to the present is never straightforward. Similarly, I highlight the significance of various religious traditions – such as Hinduism and Buddhism – to show that, even in the era of high modernism around 1900, science was never quite as modern as the reviewers seem to suggest I think.

In summary, I acknowledge that *Horizons* is largely a history of those things that we now consider as part of “modern science.” I do not, however, think that this makes my work Eurocentric in analytic terms. *Horizons* is partly a history “of” Eurocentrism, but that’s a different thing, and it is important not to confuse the two.^3^ The point of the global history of science is precisely to explode the category of what the reviewers call “a traditional and universalizing Euro-North-American vision.” *Horizons* in fact shows that the category “Euro-North-American” doesn’t make much sense, especially when it comes to the sciences. Indeed, it is already a rather revealing concatenation of identities and regions. And even within the “Euro” or the “North-American,” there was nothing “traditional” or “universal” about the sciences of past or present. Or, to paraphrase Steven Shapin (1996, p.1): there is no such thing as Western science, and *Horizons* is a book about it.

## Symmetries and asymmetries of power

The problem of Eurocentrism relates directly to another major theme addressed by the reviewers, which I will group together under the heading “power.” In this respect, I disagree quite sharply with much of the criticism. The reviewers repeatedly suggest that *Horizons* ignores or downplays a range of power relations, writing that “if more emphasis was placed on asymmetrical power relations, diffusionist narratives could be avoided.” In particular, the reviewers see a kind of naivety in how *Horizons* deals with power relations beyond European imperialism. This is especially the case, they suggest, when it comes to Indigenous peoples, postcolonial nation states, and gender. I will address each of these directly, but in summary, I think it is a mischaracterisation to suggest that *Horizons* does not sufficiently engage with these issues. Indeed, throughout my work, I engage in a kind of political version of the symmetry principle. To give one example, in the epilogue I deliberately highlight how “both” China and the United States make use of genetics as a tool of surveillance. I do not, contrary to the suggestion of the reviewers, therefore think that modern science “can only be responsibly used in the hands of Europeans and North Americans.” In fact, I don’t think any particular nation or group is more or less likely to make responsible use of science. There are no saints amongst scientists. And in *Horizons*, everyone comes off pretty badly.

Despite these disagreements, I would like to thank the reviewers for highlighting the general theme of power. It is still an area in which global history generally lacks a level of clarity.^4^ I therefore welcome the opportunity to address this. Take, for example, the place of Indigenous people in my narrative. The reviewers argue that *Horizons* “idealize[s] indigenous people as guardians of the harmony between knowledge and nature.” And they suggest that we cannot ignore “practices of exploitation and violence” amongst Indigenous people, citing the example of the Aztec Empire. I agree, and perhaps I could have done more to emphasise this. Nonetheless, the various chapters in *Horizons* that engage with Indigenous history all make pretty clear that Indigenous peoples often engaged in conflict within and between groups. I describe, for example, the inter-island violence that caused the Polynesian navigator Tupaia to flee his home and travel to Tahiti. Similarly, in the case of the Aztec Empire, which I describe as “a large, centralised, tributary state,” I highlight the imperial dimensions of mapping and the collection of natural history specimens from the Mexica side.

The reviewers raise a similar criticism when it comes to my treatment of nation states. They argue that *Horizons* presents nation states as “immovable and ahistorical figures.” They similarly suggest that I don’t really know what to do with states like Russia and Turkey, which appear “in the bag of ‘otherness,’ together with (post)colonial nations such as Mexico and India.” I think this criticism stems from a similar confusion, as with Eurocentrism, over what it means to write a history “of” something. *Horizons* is partly a history “of” nationalism and nation-building. The nation state here is an actor’s category, not the analyst’s. Throughout the second half of the book, I demonstrate how historical actors used the global networks of nineteenth- and twentieth-century science to construct – both culturally and practically – the modern nation state. It is not, therefore, that I am confused as to whether Russia is part of Europe or not. It is simply that, as a historian, I take no special view on it, but rather follow my historical actors. Prior to the eighteenth century, Russia was largely seen as a Eurasian empire in which the distinction between the “European” and the “Asian” parts did not particularly matter (Bassin, 1991). That began to change following the reforms of Peter the Great, especially following the expeditions to Siberia. By 1725, Catherine the Great could declare that “Russia is a European power” (Masoero, 2015, p.195). This vision of a “Europeanised” Russia was reinforced during the nineteenth and twentieth centuries, in part through the networks cultivated by scientists like Ilya Mechnikov and Peter Kapitsa. Even then, as the example of Ilya Mechnikov working in Odesa suggests, the exact status of the Russian nation state was continually contested, with scientists in Ukraine, amongst other regions, asserting their own identities.

The reviewers make a parallel argument when it comes to gender, suggesting that *Horizons* simply celebrates the work of female scientists “without reflecting on the conditions or contexts in which this occurred.” They then argue that “recognizing that there are different ways of being culturally, sex-gender, and socially related would enhance the reintroduction of forgotten agents to the history of science.” I agree. Whilst it is true that women and gender do not feature as consistently as I would have liked throughout the *Horizons*, this is in part a product of the very historical circumstances that I analyse. The growth of global networks of knowledge exchange from the early modern period onwards was one of the principal means through which women were excluded from the sciences, at least in formal and institutional settings. I use examples such as the German naturalist Maria Sibylla Merian, one of the very few European women to travel independently to South America in the seventeenth century, to demonstrate exceptions that effectively prove the rule. At the same time, as increasing numbers of women entered the more formal institutions of science during the nineteenth and twentieth centuries, I demonstrate the ways in which gendered notions of work created further obstacles. In doing so, I call attention to the differences between cultures of gendered scientific work, for example, in terms of expectations placed on Hindu women in postcolonial India as opposed to Russian women in the Soviet Union. Finally, when it comes to alternative visions of sex, gender, and sexuality, I address this pretty directly when it comes to genetics. In the case of the 1968 Summer Olympics, I discuss how the team of geneticists led by Alfonso Léon de Garay undertook sex determination studies of athletes which, along with other measures, underpinned the exclusion of transgender competitors. As many others have argued, the sciences weren’t just gendered. They were also the means through which gender itself was made and contested (Chiang, 2024; Fuechtner, Haynes, Jones, 2017).

## The history of science in public

I was delighted to see that the reviewers foregrounded the problem of writing the history of science for a general audience. *Horizons*, they argue, “raises questions about how historical synthesis balances accessibility with scholarly depth.” This was probably the biggest challenge of writing such a book. I was determined that *Horizons* should be a serious scholarly work – a book I hoped that academics could use in preparing their courses and that students could use to get a handle on different themes and topics. Yet on the other hand, I wanted the readership for *Horizons* to extend beyond universities and professional historians of science. That’s why I opted to publish with Penguin in the United Kingdom, who then managed the translation and regional rights. These choices then had implications for what the reviewers refer to as “the scope and limitations” of my approach to the global history of science. As they correctly identify, these different implicit audiences shaped, to an extent, my choice of narrative style and structure. My decision to begin each section with an individual – a real person – was partly intended to draw the general reader in. Similarly, my choice to organise *Horizons* according to what I call “key periods of world historical change” was partly intended to provide a clear structure for the general reader to follow.

That said, I don’t think *Horizons* would have looked very different if I had written it for a university press. The same narrative style and structure serve to reinforce a number of core historiographic points. Indeed, I developed this approach – or rather, adapted it from other historians of science – when writing my first academic monograph on the global history of phrenology for the University of Chicago Press. Foregrounding individuals, if done right, helps to highlight local contexts of materiality and practice in a way that global historians are often accused of ignoring. Similarly, a focus on individual lives can help emphasise the friction and the violence behind what might otherwise seem effortless circulation, something I discuss in the introduction to *Horizons* and have expanded upon in more recent theoretical work (Poskett, Giovannetti-Singh, 2024). Finally, a focus on individual lives immediately opens up the question of which individuals are part of the story. Is it the French astronomer Charles-Marie de La Condamine or his Peruvian Indian guides? Is it Albert Einstein or his Bengali collaborator, the physicist Satyendranath Bose?

Ultimately, when it comes to books, I don’t think there is any great tension between the scholarly and the popular. I think it is perfectly possible to write a serious academic text which makes a clear and bold argument, grounded in individual human experience, and for it to reach a wider audience. Indeed, I think it is important. As Marshall Hodgson (1993, p.255) argued over thirty years ago, asking “large-scale questions” is part of “what makes history a public discipline.” In turn, writing for general audiences forces us to develop a greater variety of “big picture” narratives, something I have argued is crucial for the future of the field (Poskett, 2024). I am grateful that the reviewers recognised this, taking seriously both the general problem of audience and the more specific intellectual challenges of writing global histories of science.

Still, reception does not always start or end with readers. And public history isn’t just about books. Since publishing *Horizons*, I’ve had the privilege to engage with a variety of audiences in different formats and media: radio, podcasts, magazines, literary festivals, and alike. I found some of this deeply rewarding and am genuinely grateful for the opportunities. At the same time, the experience of doing the history of science in public taught me a lot about what I value and what I don’t when it comes to communicating beyond academia. I also quickly learned that there are very different cultures of public engagement in different parts of the world. For example, in the United Kingdom, the media largely understands history as a form of light entertainment aimed at the chattering classes. The focus on individual lives in *Horizons*, which I thought did serious intellectual and political work, now seemed more like a series of quirky anecdotes, something to be wheeled out at a dinner party.

I was, however, relieved to learn that this was not a universal problem. In many countries, there is still a strong culture of public intellectual discussion. From what I could tell, much of continental Europe still maintains a serious culture of intellectual debate within the broader public sphere. In Italy, a radio presenter asked me about the place of Marxism in science. In Germany, a member of the public asked me about how my argument relates to Michel Foucault’s notion of power-knowledge. And in Spain, a journalist quizzed me on the specifics of the relationship between slavery, colonialism, and Newtonian science. I have less direct knowledge of how public intellectual culture functions today outside of Europe, but from a few brief encounters, I got a sense of the variety. A student based in China explained to me that there are large WeChat groups in which both academic historians and the public discuss the history of science. An online cultural magazine based in Kazakhstan similarly gave me a sense of Kazak debates on science and nationalism. During an interview, the magazine was quite willing to engage with difficult questions around what “decolonising” the history of science might mean in the context of post-Soviet states. In both these cases, increasing access to the internet over past few decades has radically changed the way in which the history of science operates in the public sphere around the world.

Nonetheless, the public sphere isn’t always a place of free and easy exchange of ideas. Censorship was something I worried about, as I didn’t have direct control over translations, nor could I easily independently verify if anything had been added or removed in foreign editions. To be fair, Penguin did a good job of trying to manage this, and they gave me a say where it was possible. Even so, I believe the edition of *Horizons* published in mainland China does omit certain passages related to genetics under Mao Zedong and the more recent persecution of the Uyghur population. (Although the Chinese edition published in Taiwan is, to my knowledge, completely uncensored.) Similarly, I was asked at one point whether I would be happy for a Russian scientist to write a preface to the Russian edition. In this case, I declined. In the wake of the Russian invasion of Ukraine, I did not want a preface written by someone who might – willingly or otherwise – reinforce Russian nationalism. Indeed, part of the point of *Horizons* was to highlight the history of Russian imperialism, as well as the place of Ukraine in the history of science, something I also wrote about subsequently elsewhere (Poskett, Shaw, 2022). To their credit, the Russian publisher respected my wishes. In the end, I made peace with all this. I knew that anyone who wanted to read the uncensored English-language edition could find it online with a bit of searching. And, in the case of Chinese readers, they could also quite easily access the uncensored Taiwanese edition.

Ultimately, I think the variety of ways that the history of science does and does not function in the public sphere is still largely underappreciated, particularly when we look beyond Europe. I thank the reviewers for raising this issue. I agree with them that “the global circulation, discussion, and coproduction of science is not only a historical question but also a current problem for science communication.” The same might be said of the history of science itself.

## Data Availability

Not deposited in a data repository.

## NOTES

1 A more general version of this question is posed by Peter Dear (2005).

2 On what constitutes “vicious anachronism” in the history of science, see Jardine (2000). For a more general discussion of “present-centred” history of science, see Timothy G. Ashplant and Adrian Wilson (1988).

3 For the history of Eurocentrism in the sciences, see what is still my favourite essay in the history of science, Elshakry (2010).

4 As made clear in the debate between Adelman (2 Mar. 2017) and Drayton and Motadel (2018).
